# Draft genome sequencing of a multidrug-resistant *Staphylococcus warneri* strain associated with endometritis in a dairy cow

**DOI:** 10.1128/mra.01007-25

**Published:** 2025-10-13

**Authors:** Mahmuda Nasrin Juthi, Tanvir Shahriar, Kh. Yeashir Arafat, Md. Naim Siddique, Md. Taimur Islam, Anup Kumar Talukder, M. Nazmul Hoque, Ziban Chandra Das

**Affiliations:** 1 Molecular Biology and Bioinformatics Laboratory, Department of Gynecology, Obstetrics and Reproductive Health, Gazipur Agricultural University 198780, Gazipur, Bangladesh; 2 Department of Pathobiology, GAU 198780 , Gazipur, Bangladesh; Fluxus Inc., Sunnyvale, California, USA

**Keywords:** *Staphylococcus warneri*, multidrug resistance, endometritis, dairy cow

## Abstract

We present the draft genome sequence of a multidrug-resistant *Staphylococcus warneri* strain, MBBL25_EM2, isolated from the uterine swab of a crossbred dairy cow with endometritis. The assembled genome of MBBL25_EM2 is 2.6 Mbp spanned over 72 contigs and harbors six antibiotic-resistance genes and 10 virulence genes.

## ANNOUNCEMENT

Endometritis is a major reproductive disease in dairy cattle, causing significant economic losses worldwide due to its negative impact on fertility and milk production (1, 2). Among the various causal agents of this disease, coagulase-negative staphylococci, including *Staphylococcus warneri*, *S. epidermidis*, *S. haemolyticus*, and *S. chromogenes,* are important contributors to bovine endometritis in cows and buffaloes (3, 4).

*S. warneri,* an opportunistic gram-positive bacterial pathogen, is increasingly acknowledged as a causative agent of infectious diseases in both humans and animals (3–5). *S. warneri* exhibits multidrug-resistant (MDR) phenomena, creating significant challenges for therapeutic management in animals (6, 7). This study presents the draft genome sequence of an MDR *S. warneri* strain, MBBL25_EM2, isolated from the uterine swab of a crossbred dairy cow diagnosed with clinical endometritis in a dairy farm from Gazipur, Bangladesh (23.99°N, 90.41°E). A uterine swab sample was collected according to the standard veterinary procedure for postpartum cows (8). Swab samples were enriched overnight in nutrient broth (Biolife, Italy) at 37°C under aerobic conditions, serially diluted up to 10^−^³, and streaked onto Mannitol Salt Agar (Oxoid, UK) for overnight incubation at 37°C (3, 6). Colonies with typical *Staphylococcus* morphology (circular, creamy white to yellow) were purified through successive subcultures (3, 4, 9). Isolate MBBL25_EM2 was presumptively identified as *Staphylococcus* spp. based on morphology, Gram staining (gram-positive cocci in clusters), and catalase, coagulase, and sugar fermentation tests (3, 10). Species-level identification of *S. warneri* was performed using the VITEK-2 system v9.01 (11). Strain MBBL25_EM2 showed resistance to colistin sulfate, nalidixic acid, tetracycline, doxycycline, ampicillin, and chloramphenicol, as determined by Kirby-Bauer disk diffusion following CLSI M100 (2023) guidelines (12). For genomic DNA extraction, a pure colony of MBBL25_EM2 was inoculated into 5 mL of nutrient broth and incubated overnight at 37°C under static (non-shaking) conditions, followed by DNA extraction using the QIAamp DNA Mini Kit (Qiagen, Germany). Whole genome libraries were prepared from 1 ng of DNA using the Nextera DNA Flex Library Preparation Kit (Illumina, San Diego, USA), and whole genome sequencing was carried out on the Illumina MiSeq platform (Illumina, USA) with a 2 × 250 bp protocol (13). Raw reads (*N *= 3,284,830) were trimmed with Trimmomatic v.0.39 (14) and quality-checked using FastQC v 0.11.7 (15). Genome assembly was performed with SPAdes v.3.15.5 (16) and annotated using the NCBI Prokaryotic Genome Annotation Pipeline v.6.10 (17). Genome completeness, antibiotic resistance genes (ARGs), virulence factor genes (VFGs), plasmids, pathogenicity, CRISPR arrays, and metabolic features were analyzed through CheckM v.1.2.4 (18), CARD v4.0.1 (19), VFDB v6.0 (20), PlasmidFinder v2.0.1 (21), PathogenFinder2 v0.5.0 (22), CRISPRCasFinder v2.0.3 (23), and RAST v2.0 server (24), respectively, with default parameters unless otherwise stated.

The features of the draft genome are presented in Table 1. The MBBL25_EM2 genome harbored 6 ARGs, 10 VFGs, 1 CRISPR array, 1 plasmid replicon, and 272 metabolic features with high pathogenicity score (0.928), indicating its significant pathogenic potential and the clinical challenge it poses in bovine endometritis.

**TABLE 1:** Genomic features of *Staphylococcus warneri* strain MBBL25_EM2

Genome feature	*S. warneri* (MBBL25_EM2)
GenBank accession	JBPUSI000000000.1
Assembled genome size (bp)	2,611,155
GC content (%)	32.5
Coverage (×)	150
Genome completeness (%)	99.2
CheckM contamination (%)	0.1
BioSample accession	SAMN49982473
SRA accession	PRJNA1291960
RefSeq	GCF_051590485.1
*N_50_* value (bp)	96149
Number of contigs	72
Genes (total)	2,598
CDSs (total)	2,541
Genes (coding)	2,496
CDSs (with protein)	2,496
Genes (RNA)	57
rRNAs	2, 1, 1 (5S, 16S, 23S)
Complete rRNAs	2, 1, 1 (5S, 16S, 23S)
tRNAs	49
ncRNAs	4
Pseudo genes (total)	45
CDSs (without protein)	45
Pseudo genes (ambiguous residues)	0 of 45
Pseudo genes (frameshifted)	14 of 45
Pseudo genes (incomplete)	23 of 45
Pseudo genes (internal stop)	17 of 45
Pseudo genes (multiple problems)	7 of 45
CRISPR arrays	1
Number of ARGs	6
Number of VFGs	10
Number of metabolic features	272
Pathogenicity score	0.928
Matched with pathogenic families	25

## Data Availability

The whole genome shotgun project of the Staphylococcus warneri strain MBBL25_EM2 has been deposited in the NCBI Sequence Read Archive (SRA accession: SRR34550965) under BioProject accession number PRJNA1291960. The assembled genome is available under accession number GCF_051590485.1, with version reported in this study being JBPUSI000000000.1.
